# Connection between the Altered HDL Antioxidant and Anti-Inflammatory Properties and the Risk to Develop Alzheimer's Disease: A Narrative Review

**DOI:** 10.1155/2021/6695796

**Published:** 2021-01-08

**Authors:** Francesca Zimetti, Maria Pia Adorni, Judit Marsillach, Cinzia Marchi, Alessandro Trentini, Giuseppe Valacchi, Carlo Cervellati

**Affiliations:** ^1^Department of Food and Drug, University of Parma, Parma 43124, Italy; ^2^Department of Medicine and Surgery, Unit of Neurosciences, University of Parma, Parma 43121, Italy; ^3^Department of Environmental & Occupational Health Sciences, University of Washington, Seattle, WA 98195, USA; ^4^Department of Biomedical and Specialist Surgical Sciences, University of Ferrara, Ferrara 44121, Italy; ^5^Plants for Human Health Institute, Animal Sciences Department, NC Research Campus Kannapolis, NC State University, 28081 NC, USA; ^6^Department of Food and Nutrition, Kyung Hee University, Seoul, Republic of Korea; ^7^Department of Morphology, Surgery and Experimental Medicine, University of Ferrara, 44121 Ferrara, Italy

## Abstract

The protein composition of high-density lipoprotein (HDL) is extremely fluid. The quantity and quality of protein constituents drive the multiple biological functions of these lipoproteins, which include the ability to contrast atherogenesis, sustained inflammation, and toxic effects of reactive species. Several diseases where inflammation and oxidative stress participate in the pathogenetic process are characterized by perturbation in the HDL proteome. This change inevitably affects the functionality of the lipoprotein. An enlightening example in this frame comes from the literature on Alzheimer's disease (AD). Growing lines of epidemiological evidence suggest that loss of HDL-associated proteins, such as lipoprotein phospholipase A2 (Lp-PLA2), glutathione peroxidase-3 (GPx-3), and paraoxonase-1 and paraoxonase-3 (PON1, PON3), may be a feature of AD, even at the early stage. Moreover, the decrease in these enzymes with antioxidant/defensive action appears to be accompanied by a parallel increase of prooxidant and proinflammatory mediators, in particular myeloperoxidase (MPO) and serum amyloid A (SAA). This type of derangement of balance between two opposite forces makes HDL dysfunctional, i.e., unable to exert its “natural” vasculoprotective property. In this review, we summarized and critically analyzed the most significant findings linking HDL accessory proteins and AD. We also discuss the most convincing hypothesis explaining the mechanism by which an observed systemic occurrence may have repercussions in the brain.

## 1. Introduction

Despite decades of intense research, the biological role of high-density lipoprotein (HDL) is still not completely understood [1, 2]. What we know up to now is that the mosaic of lipids and proteins composing its structure remarkably changes during its maturation from its precursor (formed by apolipoprotein A1 and few phospholipids) to the nascent HDL particles (pre-*β*-HDL) and, finally, to the biologically competent mature particles HDL3 and HDL2 [3]. Through this dynamic evolution process, various intermediate HDL particles are formed, and every remodeling step impacts not only its classic role in lipid transport, i.e., reverse cholesterol transport (RCT), but also other multiple functional properties. It is now well established that these HDLs are able to exert antioxidant, anti-inflammatory, antithrombotic, and immune modulation activities, acting through multiple mechanisms [4–6]. The plethora of properties endows HDL with a unique antiatherosclerotic capacity.

**Figure 1:**
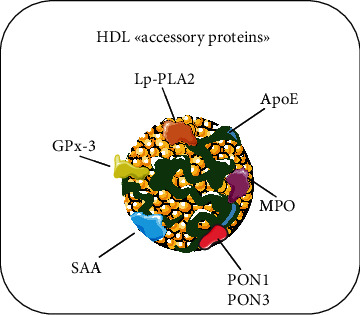
Schematic representation of HDL “accessory proteins.” The most important members of the “family of accessory proteins” associated with HDL and object of the present review are as follows: (1) myeloperoxidase (MPO); (2) serum amyloid A (SAA); (3) lipoprotein phospholipase A2 (Lp-PLA2); (4) glutathione peroxidase-3 (GPx-3) and (5) PON3.

The complex and diverse proteome (and lipidome) drives the functions of the heterogeneous family of HDLs [7]. The population of HDL particles greatly varies in protein composition and size, consisting of multiple subspecies with sizes ranging from 7 to 14 nm in diameter. Some of the constituents have been identified only recently, thanks to the implementation of mass spectrometry-based proteomic technology. The most abundant proteins in HDL are apolipoprotein A1 (ApoA1) and ApoA2, with the former present in all human HDLs and the latter in 60% of the total particles [8]. Other abundant apolipoproteins include ApoC-III, ApoE, ApoC-I, ApoC-II, and ApoM [9]. The proteome of HDL is further enriched by up to 95 accessory proteins [10], with various functions ranging from protective (antioxidant, antimicrobial, and regulation of cholesterol transport) to harmful (prooxidant, proinflammatory). These enzymes, acting in concert with apolipoproteins, contribute to the pleiotropic functions of their carrier. The most important members of these “accessory proteins” are as follows: (1) paraoxonase-1 (PON1, with antioxidant properties) [11]; (2) PON3 (also antioxidant) [12]; (3) lipoprotein phospholipase A2 (Lp-PLA2, antioxidant) [13]; (4) myeloperoxidase (MPO, prooxidant and proinflammatory) [14]; (5) glutathione peroxidase-3 (GPx-3, antioxidant) [15] and (6) serum amyloid A (SAA, prooxidant, proinflammatory) [16] (Figure 1).

It is becoming increasingly apparent that patients with clinical or subclinical cardiovascular disease (CVD) have HDL with an altered protein cargo. More specifically, the isolated particles showed decreased antioxidant-protective (e.g., PON1 and GPx-3) and increased prooxidant (e.g., MPO) proteins. This structural change inevitably alters HDL function, making it less cardioprotective. Indeed, dysfunctional HDLs are less effective in contrasting oxidation of low-density lipoproteins (LDL), occurring within the intima of the arterial wall, which renders these lipoproteins more proatherogenic.

Abundant lines of evidence suggest that circulating HDL may also provide resilience to cerebrovascular dysfunction in Alzheimer's disease (AD). Neurovascular changes have a significant and apparently precocious impact on brain metabolism and homeostasis, influencing the clearance of amyloid-*β* (A*β*) and tau protein, the precursor of the well-recognized pathological hallmarks of AD, neuritic plaques, and neurofibrillary tangles (NFT) [17–19]. The importance of the vasculature in AD is further supported by associations between CVD and AD risk and the evident overlap of many cardiometabolic risk factors [20]. This is also the case of HDL-cholesterol (HDL-C); indeed, several cross-sectional and longitudinal cohort studies have shown that high levels of HDL-C were inversely associated with the risk of developing AD, even though the results of a recent meta-analysis did not support this longitudinal association [21]. In a similar fashion, several studies investigating the cause-effect relationship between HDL-C and CVD, including the results of the trials on HDL-C-raising drugs [22, 23], have generated mixed findings, casting doubts on the cardioprotective capacity of the lipoprotein. There is now a wide consensus that these controversial data might be explained by the fact that HDL-C levels only partly determine HDLs biological functionality [24–26].

This explanation could be also true for the aforementioned findings on the relationship between HDL-C levels and AD. In support of this idea, growing population-based studies found an inverse (and independent from HDL-C plasma concentration) association between “protective” HDL accessory proteins (in particular PON1 and Lp-PLA2) and the risk of AD; at the opposite, the levels of prooxidant and proinflammatory MPO and SAA have been often found to be higher in AD compared with healthy controls. Of note is that some of the cited associations were independent of HDL-C levels. Besides, all these proteins are also expressed or found in the brain, where they seemingly influence the formation of the typical AD neuropathological traits.

In this report, we describe the opposite thinking of the most relevant laboratory and epidemiological studies related to the role that “nonapolipoprotein” components of the HDL proteome may play in AD pathogenesis. Our work is aimed at filling the gap in the literature, which lacks a critical summary on this topic, except for PON1, which has already been extensively already reviewed elsewhere [27, 28].

## 2. Myeloperoxidase (MPO)

Myeloperoxidase (MPO) is a heme protein expressed at high levels not only in neutrophils, monocytes, and certain tissue macrophages, such as those of atherosclerotic plaques and microglia, but also in neurons and endothelial cells [29, 30]. It is secreted upon leukocyte activation and plays an important role in innate immunity [29]. Once released, the active enzyme is able to catalyze the oxidation of halides and pseudohalides in the presence of hydrogen peroxide (H_2_O_2_). This mild reactive oxygen species (ROS) is generated from the dismutation of superoxide anion, which in turn is mostly provided by the nicotinamide adenine dinucleotide phosphate oxidase (NOX) 2 system, enriching membranes of activated granulocytes. The most important oxidizing agents formed by MPO are hypochlorous acid (HOCl) and hypothiocyanous acid (HOSCN), which contribute to the elimination of microorganisms as well as aberrant mammalian cells. Besides, once degranulated, a large amount of MPO remains inactive, playing an important role in the induction of cytokines. As a matter of fact, MPO in this role is beneficial to the host [31].

MPO-derived reactive species preferentially and indiscriminately target lipids and proteins of nonself, but also self, biological entities. As a logical consequence, inflammation-induced excess activation of MPO could cause damage to any tissue/cell. These adverse effects account for the widely documented association between elevated circulating levels of MPO and “classical” inflammatory responses such as those detected in rheumatoid arthritis, Crohn's disease, and CVD [29, 32–34]. In particular, many clinical reports showed a strong link between MPO and a wide variety of CVDs, with increased blood MPO levels being associated with poor prognosis in affected patients.

One of the most harmful effects of abnormal activation of MPO is the oxidation of proteins and lipids carried by circulating HDL and LDL. This particular chemical transformation scales up the magnitude of the MPO's impact because toxic ox-LDLs and dysfunctional HDLs can spread from the site of transformation to others throughout the body. The oxidative challenge of this leukocyte-derived enzyme is facilitated by its physical association with ApoA1 and HDL particles [14]. Intriguingly, it has been hypothesized that MPO and PON1 mutually modulate each other, the latter attenuating the prooxidant activity of the former, which, in turn, is able to impair the function of both PON1 and ApoA1. Accordingly, increased nitration and chlorination has been observed in ApoA1 isolated from patients affected by CVDs. Evidence indicates that MPO-induced modification of specific residues on this apolipoprotein may affect the cholesterol efflux capacity, the lecithin-cholesterol acyltransferase (LCAT) activity and the anti-inflammatory, antioxidant and antiapoptotic properties of HDL. In other words, MPO represents the major culprit of the shift from an anti- to a proatherogenic HDL phenotype.

### 2.1. Myeloperoxidase in Alzheimer's Disease

MPO is also produced within the brain tissue by microglia and neutrophils that infiltrate the Central Nervous System (CNS) during the neuroinflammatory process. Brain inflammation is an important component of the pathogenesis of AD; thus, it is not surprising that the postmortem neocortex, hippocampus, and temporal cortex of AD patients present higher amounts of MPO protein compared with those of controls. In these brain areas affected by neurodegeneration, MPO colocalizes with A*β* in senile plaques. Neuroinflammation and oxidative stress (OxS) in AD can be triggered by stroke, the most common presentation of cerebrovascular disease that contributes to AD neuropathological changes. Using an enzyme-activated MRI agent that can track the oxidative activity of MPO [35], it has been shown that MPO accumulates in large amounts also during ischemia [35, 36].

**Figure 2:**
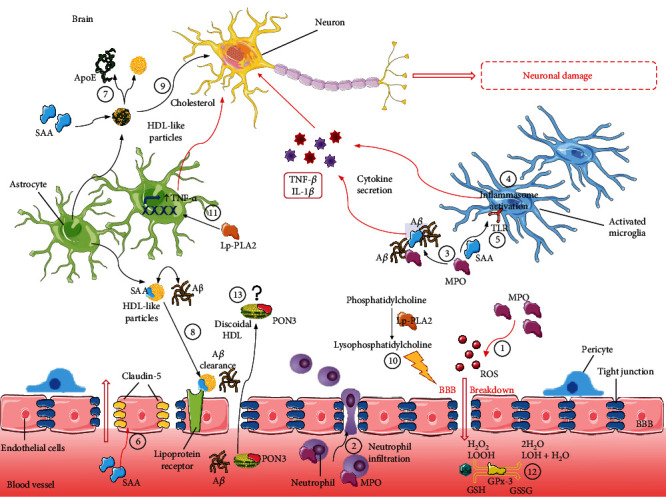
Role of HDL accessory proteins in AD physiopathology. Myeloperoxidase (MPO) participates in BBB breakdown by inducing the production of ROS (1) or by increasing neutrophil recruitment through interaction with the brain endothelium (2). MPO and serum amyloid A (SAA) may interact with A*β* plaques, inducing cytokine release and thus exacerbating neuroinflammation (3). In the CNS, SAA can activate glial cells, inducing the secretion of inflammatory cytokines through the activation of the inflammasome (4) and the modulation of the Toll-like receptors (TLR) (5). Moreover, SAA could directly contribute to the BBB breakdown by decreasing the expression of claudin-5, one of the tight junction components (6). SAA may induce the dissociation of apolipoprotein E (ApoE) from HDL-like particles present in the cerebrospinal fluid (CSF) (7), producing particles less able to bind A*β* and thus to mediate its clearance through the lipoprotein receptors (8). SAA enrichment could also affect CSF HDL function by interfering with their functions in mediating the brain cholesterol transport, essential to provide cholesterol to neurons (9). Lipoprotein phospholipase A2 (Lp-PLA2) produces phosphatidylcholine by enzymatic hydrolysis, a mediator of inflammatory stress in brain endothelial cells, increasing endothelial permeability, thus potentially affecting BBB integrity (10). Moreover, Lp-PLA2 activity can promote the expression of TNF-*α*, a key cytokine responsible for increased neuroinflammation (11). The decrease of selenium bioavailability could affect the glutathione peroxidase-3 (GPx-3) activity, negatively influencing the antioxidant defense mechanisms implicated in the removal of H_2_O_2_ and neutralization of lipid peroxides (12). The role of paraoxonase-3 (PON3) in AD is still under investigation, and the hypothesis is that it is produced by the liver and reaches the CNS by crossing the BBB in discoidal HDLs via an unknown mechanism [38] (13), as also hypothesized for PON1 [27, 28].

It is well known that under inflammatory conditions, the blood-brain barrier (BBB) function is compromised and can aggravate neuronal dysfunction in AD patients. Experiments conducted *in vitro* and *in vivo* by Ullen and coworkers revealed that MPO participates in BBB breakdown by releasing oxidant species or, indirectly, by increasing inflammatory cells (neutrophils) recruitment via electrostatic interactions of MPO with the brain endothelium (Figure 2). The authors concluded that MPO inhibitors could be effective in protecting BBB integrity and, thereby, favorably in interfering with disease progression. Notably, the same optimistic conclusions about the possible use of MPO inhibition in AD treatment have been recently drawn in a study on an AD animal model (5XFAD mice) [37].

A possible association between increased MPO and AD has been also reported in some population-based studies, although results were not univocal, leaving still open the question (Table 1). More specifically, we found two studies reporting higher levels of this enzyme in the plasma of AD patients compared with cognitively normal subjects [39, 40] and two studies with no differences detected [41, 42]. Relevant to this context, Tzikas et al. found that the association of MPO levels with the presence of AD was not the mere result of the influence that this enzyme has on the classical CVD risk factors, such as diabetes, hypertension, and obesity, among others. [39]. Indeed, the study showed that the enzyme was positively correlated with the plasma A*β*_1-42/1-40_ ratio, which has been found to be a potential predictor for the development of cognitive decline preceding AD [43].

In agreement with the potential link between MPO and AD, some genetic investigations on *MPO* polymorphisms found a link with genotypes associated with elevated protein expression and disease susceptibility [56–59].

The potential key role of MPO in the pathogenesis of several OxS and inflammation-related disorders has considerably raised the interest in the development and clinical use of MPO inhibitors. Many reversible and irreversible inhibitors had been employed in *in vivo* disease models, with some of them showing promising outcomes. One of the most solid results in this context was obtained by Yu et al., who showed that a synthesized peptide blocking MPO activity was able to reduce brain damage in murine models of stroke and to restore BBB integrity [60]. However, to the best of our knowledge, no MPO inhibitor has still been tested in clinical trials. The main issue limiting this type of approach is that downmodulating MPO activity, on one hand, successfully attenuates the reactive species-induced tissue damage and, on the other hand, negatively affects its essential role in host defense.

## 3. Serum Amyloid A (SAA)

Serum amyloid A (SAA) consists of a family of proteins that share a high degree of homology, encoded by genes highly conserved throughout evolution. In humans, there are 4 SAA genes, but only *saa1* and *saa2* encode acute phase proteins, highly inducible during the acute phase response [61, 62]. Due to its high hydrophobicity, only a small fraction of SAA is present in plasma in a lipid-free form. SAA is in fact normally associated with lipoproteins, mainly HDL [63], but it may also be bound to ApoB-containing particles through the activity of the cholesteryl ester transfer protein (CETP) [64]. The liver is the main site of SAA production, although stimulated monocytes/macrophages, vascular smooth muscle cells, and endothelial cells may locally secrete SAA during chronic inflammatory conditions. Plasma SAA concentrations can rise up to 1000-fold within 24 h when stimulated by the acute phase response, while modest increases of SAA levels (+10-fold) usually accompany chronic inflammatory disorders such as atherosclerosis [65].

SAA is strictly linked to atherosclerosis and CVD and increased SAA is associated with CVD mortality, at least from epidemiological studies. In this regard, elevated SAA1 plasma levels have been recently suggested to accurately individuate the presence of acute coronary syndrome (ACS), correlating with the severity of coronary artery disease (CAD) in the studied patients [66]. Conversely, other authors reported a lack of association between SAA levels and the incidence of ACS in a cohort of 167 patients, with SAA marginally linked to the odds of unstable angina, taken as a secondary endpoint [67]. A more persuasive result on the relationship between SAA and CVD comes from the results of a recent meta-analysis of 26 studies in which authors found a significant association between SAA and the increased risk of coronary heart disease (CHD), with SAA levels correlating with the disease severity [68]. This link was particularly significant in subjects aged more than 55 years old, from European and Asian origin, or belonging to case-control studies.

Although the pathophysiological link explaining the association between SAA, atherosclerosis, and CVD is still far from being clarified, there may be multiple mechanisms involved, among which inflammation exacerbation would play a key role. SAA acts as a cytokine-like protein by regulating several cell inflammatory responses, inducing, for example, the release of cytokines and the activation of the NLRP3 inflammasome [61]. Other mechanisms include increased aberrant proliferative capacity of smooth muscle cells, induction of chemotaxis, and migration of monocytes into the atherosclerotic lesions, endothelial dysfunction and changes in HDL composition and function [61, 69].

As mentioned above, plasma SAA is lipoprotein-associated and evidence indicates that this association is a critical factor modulating its activity. In particular, evidence from *in vitro* studies suggests that SAA association to HDL limits its proinflammatory action [70] because of the entrapment of SAA-enriched HDL by proteoglycans [71] in both macrophages and adipocytes. This retention is mediated by the presence of specific proteins [71].

Thus, HDL enrichment in SAA profoundly alters the composition and structure of HDL, impacting its antioxidant/anti-inflammatory function, as well as its capacity to promote cholesterol efflux [72]. The latter is a functional property representing the ability of these lipoproteins to remove excess cholesterol from the arterial wall, thus worsening lipid deposition in plaques [8]. This negative impact on HDL function seems related to the capacity of SAA to displace ApoA1 from HDLs [73]. On the other way around, HDLs are able to inhibit SAA proinflammatory, prooxidant and prothrombotic activities, as emerged from *in vitro* and preclinical studies [65, 74].

### 3.1. Serum Amyloid A in Alzheimer's Disease

The prooxidant and proinflammatory activity of SAA may also occur in the CNS, possibly playing a role in cognitive decline and AD pathogenesis. In recent studies in mild cognitive impairment (MCI) and cognitively healthy subjects, it was found that plasma SAA levels were lower in the control group and gradually increased from MCI to AD patients [44, 45]. In addition, SAA levels inversely correlated with the Mini-Mental State Examination (MMSE) score [44], suggesting SAA as an early biomarker of cognitive impairment (Table 1).

More specifically, SAA1 produced by the liver seems to accumulate in mouse and human AD brains, as reported in previous studies [16, 75]. Liver-derived SAA1 is thus able to reach the CNS where it can activate the glial cells and induce the secretion of inflammatory cytokines, the activation of the inflammasome and the modulation of the Toll-like receptors [76] (Figure 2). Based on these studies, it is likely that SAA1 may exacerbate neuroinflammation, and its reported interaction with A*β* deposits [16] strengths the hypothesis of its involvement in AD pathogenesis. The link between SAA1 and A*β* is clearly explained by the results of a recent *in vivo* study, in which AD mouse models overexpressing SAA1 showed increased inflammation, documented by glial cell activation and increased release of cytokines, augmented amyloid aggregation, and worsened memory decline [77].

In addition, SAA may influence other important cerebral processes involved in AD pathophysiology by interfering, for example, with the BBB permeability (Figure 2). In this regard, higher SAA levels in serum and CSF have been observed in patients with BBB integrity impairment compared with those with intact BBB among subjects aged 55 or older [78] (Table 1). Thus, increased BBB permeability may allow a higher accumulation of cerebral SAA, thus triggering the inflammatory response and leading to brain dysfunction. Actually, SAA was also found to directly damage the BBB *in vitro* by decreasing the expression of claudin-5, a component of tight junctions [79]. Such damage was abrogated by the coincubation of cells with both SAA and HDL, underlying the importance of the balance between these two molecules in the maintenance of BBB integrity.

Concerning the relationship between SAA and HDL in the brain, previous work demonstrated that SAA levels are increased by about 20-fold in the CSF of AD patients [16] (Table 1). More specifically, SAA induces the dissociation of ApoE from the HDL-like particles present in the CSF, as occurs for ApoA1 from plasma HDL. The loss of ApoE leads to particles less able to bind A*β* and thus to mediate its clearance through the lipoprotein receptors [80] (Figure 2). Moreover, as a result of reduced ApoE-bound particles, normally large and spherical, elevated SAA can affect CSF HDL function, such as the capacity to mediate the brain cholesterol transport, essential to provide cholesterol to neurons [81]. Consistently, we have previously demonstrated that CSF from AD patients is less able to promote cholesterol efflux through the membrane transporter ATP-binding cassette G1 (ABCG1), which in the brain mediates efflux to ApoE-enriched large particles [82].

Based on the described SAA role in AD, a potential effective therapeutic approach could target the SAA receptor promoting the SAA cell internalization and thus its proinflammatory activity [83]. In this regard, a nanoparticle-based approach targeting SR-BI, the scavenger receptor for HDLs, has been proposed by some authors to counteract SAA's deleterious effects [84]. Another suggestion for a potential therapeutic strategy comes from *in vitro* evidence. It was found that co-ultramicronized palmitoylethanolamide/luteolin, an agent with neuroprotective properties [85], significantly attenuated the cytokine tumor necrosis factor-α (TNF-*α*)-induced increase in *Saa1* gene expression in oligodendrocyte precursor cells [86]. These observations need to be further investigated in appropriate preclinical models.

## 4. Lipoprotein Phospholipase A2 (Lp-PLA2)

Lipoprotein phospholipase A2 (Lp-PLA2), also known as platelet-activating factor acetylhydrolase (PAF-AH), is a member of the superfamily of PLA2 enzymes, which play an important role in redox processes, inflammation, and atherosclerosis. Lp-PLA2 is mainly secreted by macrophages and circulates in the blood complexed with LDL and, to a minor extent, HDL [11].

The catalytic mechanism and physiological/pathological role of Lp-PLA2 are still not clear [87–89]. This protein catalyzes the hydrolysis of the acetyl group at the sn-2 position of platelet-activating factor (PAF), thereby inactivating this proinflammatory phospholipid (PL). The enzyme also seems to be able to hydrolyze oxidized phospholipids (ox-PL) with a chemical structure similar to that of its natural endogen substrate. Some of these modified lipids are potent prooxidant and proinflammatory agents. Indeed, they can trigger the recruitment of macrophages and contribute to the initiation and progression of the inflammatory response in atherogenesis. Moreover, the inactivation of ox-PL may attenuate the proinflammatory activity of ox-LDL [90].

Despite the apparently beneficial impact of Lp-PLA2, its association with atherosclerosis is ambiguous, as it can both degrade or generate potentially damaging vasoactive molecules. The major products of enzymatic hydrolysis are lysophosphatidylcholine (LPC) and oxidized and/or nonoxidized nonesterified fatty acids (NEFA). Elevated concentration of LPC is associated with vascular damage, inflammation and atherosclerosis. On the other hand, the oxidized NEFA produced by the enzyme play multiple roles, including vasoprotective and antiatherogenic activities. A dual pro- and anti-inflammatory/antioxidant role depending on the concentration, the availability of potential substrates and the binding to lipoproteins (HDL-bound enzyme seems to be more protective than the LDL-bound enzyme) had been suggested to explain this apparent schizophrenic behavior.

A most recent meta-analysis has shown that greater Lp-PLA2 activity or mass was independently associated with increased risk of stroke and CV events in patients with stable CHD [13]. Prior to these studies, a systematic review on 32 prospective investigations demonstrated that the associations of Lp-PLA2 are not exclusive to vascular outcomes, and the vascular associations depend, at least partly, on plasmatic lipids (mainly non-HDL-C) [91]. Conversely, other studies on the topic showed no relationship with CVD risk.

The multifaceted effects of Lp-PLA2 may account, at least in part, for the inconsistency of the epidemiological results concerning the relationship of plasma enzymatic activity and concentration levels with CVD.

### 4.1. Lipoprotein Phospholipase A2 in Alzheimer's Disease

The potential proinflammatory and proatherogenic role of Lp-PLA2 was the major rationale for several epidemiological studies on the association between activity/mass of this enzyme and dementia. Considering its nature, Lp-PLA2 could be a static biomarker of AD risk, essentially reflecting predisposition and/or underlying physiopathological conditions of AD, such as low-grade inflammation, atherosclerosis, and OxS. However, this enzyme also has the potential to directly contribute to the pathogenic process of AD. Indeed, even in the absence of direct evidence and solid mechanistic insights, it has been demonstrated that products of its hydrolytic activity may play an active role in this context. Indeed, LPC is a mediator of inflammatory stress on brain microvascular endothelial cells [92] and increases the permeability of endothelial cells, thus potentially affecting BBB integrity [93]. Moreover, Lp-PLA2 activity can promote the expression of TNF-*α*, which is a key cytokine affecting hippocampal neuroplasticity (Figure 2).

The first large population-based study (*n* = 6713), showing that subjects with higher levels of Lp-PLA2 activity were at increased risk of all-cause dementia, was published in 2005 (Table 1). The relationship was independent of inflammatory markers and CVD risk factors, concluding that the role of Lp-PLA2 in systemic inflammation and atherosclerosis could not completely explain its association with CNS disorders [46]. Notably, the authors found that Lp-PLA2 activity was not significantly associated with vascular dementia (i.e., the second most frequent form of dementia) and AD, although effect estimates were distinctly stronger for the former. Similar results have been shown in a more recent report from the Cardiovascular Health Study, where the risk of AD was increased twofold in the highest compared to the lowest quartile of Lp-PLA2 mass [47]. In contrast with these two and with other cross-sectional studies [48, 49], the Framingham Heart Study was unable to associate Lp-PLA2 with risk for dementia or with microbleeds as indicators of cerebral amyloid angiopathy [50, 94]. Consistently, Davidson et al. reported no change in the enzyme activity in amnestic MCI compared to cognitively healthy subjects [95] (Table 1).

Differently, Cai et al. have recently suggested that elevated Lp-PLA2 levels may be linked to cognitive decline through the vascular pathology in individuals already presenting a state of chronic systemic inflammation. This hypothesis is supported by data showing that higher Lp-PLA2 mass/activity is an independent risk factor for MCI in type 2 diabetes mellitus patients. In light of these observations, Lp-PLA2 might represent a central player in the vicious circle leading to the accumulation of inflammatory cells and cytokines and vascular deficit [96].

From our point of view, a scenario where elevated Lp-PLA2 brings a major susceptibility to AD only in certain (predisposed) subpopulations is plausible. A further argument in support of this hypothesis comes from studies on diabetic and hypercholesterolaemic pig models. Treatment with darapladib (Lp-PLA2 inhibitor) appeared to reduce BBB leakage and to significantly lower the total amount of brain A*β*_1–42_ deposition in treated compared with untreated animals [97]. These findings were the source of inspiration for a successive randomized, double-blind, placebo-controlled study on subjects with possible mild AD and with neuroimaging evidence of cerebrovascular disease [98]. Treatment with the Lp-PLA2 inhibitor slowed the progression of AD and improved disease-associated biomarkers. Obviously, this was a mere exploratory study, and as also acknowledged by the authors, the findings require replication and extension in longer-term clinical trials.

## 5. Glutathione Peroxidase-3 (GPx-3)

GPx is the general term for a family of several selenium- (Se-) dependent isozymes that use reduced glutathione (GSH) as an obligate cosubstrate in the reduction of hydrogen peroxide to water [99, 100]. Eight mammalian isoenzymes are known, with the intracellular and ubiquitous GPx-1 and GPx-4, the gastrointestinal GPx-4, and the plasma GPx-3 being the most abundant ones. All members of the GPx family play, although to a different extent, a crucial role in the intracellular antioxidant defensive mechanism [100]. Indeed, besides the removal of mild reactive H_2_O_2_ (mostly derived from the dismutation of superoxide produced in mitochondria), they can neutralize lipid hydroperoxides and halt the deleterious peroxidation of cell membranes [100].

As mentioned above, GPx-3 is the main form detectable in plasma, but it is also present in the lung tissue, kidney (the source of its plasma secretion), and adipose tissue, as well as, at lower levels, in other tissues and in the surrounding extracellular environment [101]. From the site of production, GPx is secreted into the surrounding extracellular environment. The regulation of *GPx*-*3* gene transcription seems to vary between cell types and tissues. Irrespective of the biological context, this gene is upregulated in inflammatory conditions likely as a result of the associated redox dyshomeostasis [101].

GPx-3 has been found bound to HDL in human plasma, and its lipid peroxide-reducing activity might be important in protecting the endothelial cells as well as circulating LDL from oxidation [102]. This role might account for several studies showing altered levels of GPx-3 expression and/or serum activity in cancer [103] and CVD [104, 105]. However, the real impact of this enzyme on the extracellular defensive mechanism has not been satisfactorily clarified. The major doubts in this regard come from the relatively low availability of GSH in plasma (around 30 *μ*M), which is almost 1000 times lower than in cytosol [106]. The limited extracellular availability of the substrate might significantly affect the antioxidant capacity of the GPx isoenzyme. Consistently, it has been suggested that GPx-3 activity could be effective only in specific sites (hepatic vein plasma, epithelial cells of the gastrointestinal tract) where GSH is more abundant [106]. Alternatively, under the condition of GSH deficiency, GPx-3 might be able to use glutaredoxin and thioredoxin reductase by itself or with thioredoxin as alternate electron donors to GSH [107].

Besides GSH, the plasma concentration of selenium (Se) is a pivotal regulator of GPx-3 activity. It is well known that the circulating Se in plasma essentially consists of the extracellular Selenoproteins, GPx-3, Selenoprotein P (SELENOP), and nonspecific protein forms such as selenomethionine [108]. Both GPx-3 and SELENOP are considered biomarkers of Se bioavailability, and their levels appear to be influenced by dietary intake of this trace element [108]. In the last two decades, there has been growing interest in the implication of Se in health and disease. It is now well established that Se deficiency is the major etiopathogenic factor of two rare diseases, Kashin-Beck disease and Keshan disease. Besides, this condition was found to be associated with an increased risk of various chronic and systemic diseases [109].

### 5.1. Glutathione Peroxidase-3 and Alzheimer's Disease

The possible association between GPx-3 levels and AD has been investigated in several studies dealing with the evaluation of a panel of peripheral OxS biomarkers [110]. The published observational data reported unvaried [51, 111] or lower [15, 53, 55, 112] levels of this enzyme in AD patients compared with controls and are summarized in Table 1. In one of the largest population-based studies, Rinaldi et al. found that the activity level of GPx-3 was similarly lower in MCI (*n* = 25) and AD (*n* = 63) patients as compared with controls (*n* = 53) [54]. A similar trend was observed by Torres et al. [113] and Casado et al. [53], with the latter also showing a decrease in other antioxidant enzymes, such as superoxide dismutase and catalase. On the contrary, one large study (*n* = 338) found that, in AD, the increase in oxidative damage levels was not accompanied by a significant decrease in GPx-3 between controls and AD [52]. As stated in a recent systematic review [110], this apparent inconsistency could be due to the small sample size or different patient characteristics among the studies.

Some studies reported that a decrease of GPx-3 protein and activity should be framed in a picture of systemic redox imbalance characterizing AD. However, it cannot be ruled out that this change may merely (and statically) reflect the parallel decrement in plasma levels of Se. Consistently, a recent meta-analysis has shown that circulatory Se concentration is significantly lower among AD patients as compared to controls. This decreased bioavailability of Se affects not only the activity of the extracellular GPx but also the intracellular content of GPx-1 and GPx-4. Due to the abundant cytosolic content of GSH, these two isoenzymes have a higher antioxidant capacity compared with GPx-3, and thus, the decrease in their catalytic efficiency can greatly influence the antioxidant defensive mechanism. To the best of our knowledge, potential pharmacological strategies that are aimed at modulating the expression or activity of this specific protein in the context of neurodegenerative diseases have not been investigated yet. On the contrary, there are a number of preclinical studies investigating the effect of the positive modulator of GPx-3 in cancer cells that exhibit the ability to inhibit the proliferation and metastatic behavior [103].

## 6. Paraoxonase-3 (PON3)

Paraoxonase-3 (PON3) was the last member of the paraoxonase family to be described and, therefore, the least characterized. PON3 is also a potent antioxidant, anti-inflammatory, and antiapoptotic calcium-dependent enzyme, synthesized mainly in the liver and kidney, and found in circulation in HDLs [12], as well as in the endoplasmic reticulum of intestinal cells [114] and in mitochondria of certain tissues [115]. PON3 protein expression has also been reported in almost all examined murine tissues, together with PON1 [116]. Although PON3 concentration in circulation is much lower than PON1, about 2 orders of magnitude, it has been shown to be a more potent antioxidant than PON1 *in vitro* [12]. Unlike PON1, PON3 cannot hydrolyze organophosphate compounds, but it hydrolyzes statin lactones at a much higher catalytic efficiency than PON1 [117]. PON3 has been shown to play a protective role in CVD [118], obesity [119], and innate immunity [120] and has an oncogenic role in cancer [115].

PON3 presents a number of polymorphisms in its gene. A few studies have focused on polymorphisms in the *PON3* gene but have mostly failed to show an effect on PON3 activity, concentration, or disease [121–124]. Changes in plasma/serum PON3 concentration or activity have been studied in a variety of oxidative stress-related diseases. Increased serum PON3 protein concentration has been reported in chronic liver disease [125], HIV infection [126], and atherothrombotic disease [127] and in patients with sepsis [128], while a decrease of PON3 concentration has been detected in certain autoimmune diseases [129].

### 6.1. Paraoxonase-3 and Alzheimer's Disease

To date, there is only one study related to PON3 in AD, which involved haplotype associations between several SNPs in the entire *PON* gene (including *PON1*, *PON2*, and *PON3*) in a cohort of Caucasian and African Americans [123]. They reported some haplotype associations between polymorphisms in the *PON* gene and AD, but the most significant association was found in one *PON1* SNP in the promoter region. Nonetheless, PON3 is another potent antioxidant enzyme that, similar to PON1, is also present in both plasma/serum and CSF (Marsillach et al., unpublished data) and has documented protein localization in the brain [116]. We hypothesize that the PON3 present in CSF was originally synthesized in the liver and crosses the BBB in discoidal HDLs via an unknown mechanism, as also hypothesized for PON1 [28] (Figure 2). Altogether, PON3 could play an important role both in the CSF and in certain brain areas in the pathogenesis of neurodegenerative diseases such as AD, and more research in the near future will hopefully be pursued in this highly unexplored PON3 field.

Given the potential important role of PON3 in oxidative stress-related diseases, including AD, the study of factors that modulate the activity and/or expression of PON3 would be of great interest as potential future pharmacological treatments. No information in this regard is currently available. However, based on the significant homology of the 3 paraoxonases, we could speculate that factors known to modulate PON1 and PON2 would likely have an effect on PON3. There are excellent reviews related to factors that modulate PON1 and PON2 [130, 131]. For instance, dietary antioxidants, such as the polyphenol quercetin, which have been shown to have a dramatic impact on PON1 and PON2 activity, could potentially also increase PON3 activity. Interestingly, there are extensive studies on the neuroprotective effects of quercetin in AD [132–134]. Another approach to increase levels of PON3 could be the direct administration of exogenous PON3. Both strategies, administration of a pharmacological factor or exogenous PON3, should be considered in future studies as they can potentially lead to beneficial clinical outcomes.

## 7. Conclusion

The present review has systematically examined the most relevant data on some important “non-apolipoprotein” components of HDL and their involvement in AD pathogenesis, focusing on myeloperoxidase, serum amyloid A, lipoprotein phospholipase A2, glutathione peroxidase-3, and paraoxonase-3. Although the available clinical studies are still limited, evaluating a small or moderate number of subjects and mostly cross-sectional, mounting evidence indicates how the oxidative and inflammatory imbalance affect HDL properties, possibly playing a role in AD pathogenesis and progression of the disease. The mechanisms involved include an indirect effect on the classical cardiovascular risk factors implicated in AD development or a more direct impact on specific AD pathogenic processes, like the BBB integrity, the cerebral A*β* deposition, or other pathways. With respect to PON3, its association with AD is still largely unexplored compared to the other HDL-associated proteins and needs further investigation.

In conclusion, in light of the available data discussed in the present review, the HDL-associated proteins are certainly a potential player in AD. Future directions may include prospective studies to evaluate the impact of these HDL components on AD incidence in order to identify novel HDL-associated potential pharmacological targets, as well as pharmacological treatments to influence the expression of these HDL accessory proteins.

## Figures and Tables

**Table 1:** Association between plasma levels of myeloperoxidase, serum amyloid A, lipoprotein phospholipase A2, glutathione peroxidase-3 and the presence or the risk of Alzheimer's disease (AD), as reported in selected epidemiological studies.

Study	Study design	*n* ^∗^	AD vs. controls
(i) Myeloperoxidase			
Tzikas et al. [39]	Cross-sectional	55	↑
Cheng et al. [40]	Cross-sectional	199	↑
Marksteiner et al. [41]	Cross-sectional	56	↔
Wu et al. [42]	Cross-sectional	170	↔
(ii) Serum amyloid A			
Cao and Chen [44]	Cross-sectional	352	↑
Shang et al. [45]	Cross-sectional	673	↑
Kindy et al. (CSF) [16]	Cross-sectional	31	↑
(iii) Lipoprotein phospholipase A2			
van Oijen et al. [46]	Longitudinal	6713	↑
Fitzpatrick et al. [47]	Longitudinal	3320	↑
Bacchetti et al. [48]	Cross-sectional	83	↑
Doody et al. [49]	Cross-sectional	395	↑
van Himbergen et al. [50]	Longitudinal	541	↔
(iv) Glutathione peroxidase-3			
Ceballos-Picot et al. [51]	Cross-sectional	74	↔
Serra et al. [52]	Cross-sectional	192	↔
Puertas et al. [15]	Cross-sectional	92	↓
Casado et al. [53]	Cross-sectional	150	↓
Rinaldi et al. [54]	Cross-sectional	141	↓
Vural et al. [55]	Cross-sectional	100	↓

## References

[1] Rosenson R. S., Brewer H. B., Ansell B. J. (2016). Dysfunctional HDL and atherosclerotic cardiovascular disease. *Nature Reviews Cardiology*.

[2] Camont L., Chapman M. J., Kontush A. (2011). Biological activities of HDL subpopulations and their relevance to cardiovascular disease. *Trends in Molecular Medicine*.

[3] Gugliucci A., Menini T. (2015). Paraoxonase 1 and HDL maturation. *Clinica Chimica Acta*.

[4] Cervellati C., Vigna G. B., Trentini A. (2019). Paraoxonase-1 activities in individuals with different HDL circulating levels: implication in reverse cholesterol transport and early vascular damage. *Atherosclerosis*.

[5] Mc Manus J., Mc Eneny J., Thompson W., Young I. S. (1997). The effect of hormone replacement therapy on the oxidation of low density lipoprotein in postmenopausal women. *Atherosclerosis*.

[6] Mineo C., Shaul P. W. (2012). Novel biological functions of high-density lipoprotein cholesterol. *Circulation Research*.

[7] Kontush A., Lindahl M., Lhomme M., Calabresi L., Chapman M. J., Davidson W. S. (2015). Structure of HDL: particle subclasses and molecular components. *Handbook of Experimental Pharmacology*.

[8] Ouimet M., Barrett T. J., Fisher E. A. (2019). HDL and reverse cholesterol transport. *Circulation Research*.

[9] Shao B., Heinecke J. W. (2018). Quantifying HDL proteins by mass spectrometry: how many proteins are there and what are their functions?. *Expert Review of Proteomics*.

[10] Melchior J. T., Street S. E., Andraski A. B. (2017). Apolipoprotein A-II alters the proteome of human lipoproteins and enhances cholesterol efflux from ABCA1. *Journal of Lipid Research*.

[11] Passaro A., Vigna G. B., Romani A. (2018). Distribution of paraoxonase-1 (PON-1) and lipoprotein phospholipase A2 (Lp- PLA2) across lipoprotein subclasses in subjects with type 2 diabetes. *Oxidative Medicine and Cellular Longevity*.

[12] Draganov D. I., Stetson P. L., Watson C. E., Billecke S. S., La Du B. N. (2000). Rabbit serum paraoxonase 3 (PON3) is a high density lipoprotein-associated lactonase and protects low density lipoprotein against oxidation. *The Journal of Biological Chemistry*.

[13] Li D., Zhao L., Yu J. (2017). Lipoprotein-associated phospholipase A2 in coronary heart disease: review and meta-analysis. *Clinica Chimica Acta*.

[14] Huang Y., Wu Z., Riwanto M. (2013). Myeloperoxidase, paraoxonase-1, and HDL form a functional ternary complex. *The Journal of Clinical Investigation*.

[15] Puertas M. C., Martínez-Martos J. M., Cobo M. P., Carrera M. P., Mayas M. D., Ramírez-Expósito M. J. (2012). Plasma oxidative stress parameters in men and women with early stage Alzheimer type dementia. *Experimental Gerontology*.

[16] Kindy M. S., Yu J., Guo J.-T., Zhu H. (1999). Apolipoprotein serum amyloid A in Alzheimer’s disease. *Journal of Alzheimer’s Disease*.

[17] Cervellati C., Trentini A., Pecorelli A., Valacchi G. (2020). Inflammation in neurological disorders: the thin boundary between brain and periphery. *Antioxidants & Redox Signaling*.

[18] De La Torre J. C. (2004). Is Alzheimer’s disease a neurodegenerative or a vascular disorder? Data, dogma, and dialectics. *The Lancet Neurology*.

[19] Iturria-Medina Y., Sotero R. C., Toussaint P. J., Mateos-Pérez J. M., Evans A. C. (2016). Early role of vascular dysregulation on late-onset Alzheimer’s disease based on multifactorial data-driven analysis. *Nature Communications*.

[20] Button E. B., Robert J., Caffrey T. M., Fan J., Zhao W., Wellington C. L. (2019). HDL from an Alzheimer’s disease perspective. *Current Opinion in Lipidology*.

[21] Anstey K. J., Ashby-Mitchell K., Peters R. (2017). Updating the evidence on the association between serum cholesterol and risk of late-life dementia: review and meta-analysis. *Journal of Alzheimer’s Disease*.

[22] Boden W. E., Probstfield J. L., Anderson T. (2011). Niacin in patients with low HDL cholesterol levels receiving intensive statin therapy. *The New England Journal of Medicine*.

[23] Lincoff A. M., Nicholls S. J., Riesmeyer J. S. (2017). Evacetrapib and cardiovascular outcomes in high-risk vascular disease. *The New England Journal of Medicine*.

[24] Pappa E., Elisaf M. S., Kostara C., Bairaktari E., Tsimihodimos V. K. (2020). Cardioprotective properties of HDL: structural and functional considerations. *Current Medicinal Chemistry*.

[25] Vigna G. B., Satta E., Bernini F. (2014). Flow-mediated dilation, carotid wall thickness and HDL function in subjects with hyperalphalipoproteinemia. *Nutrition, Metabolism, and Cardiovascular Diseases*.

[26] Ossoli A., Pavanello C., Giorgio E., Calabresi L., Gomaraschi M. (2019). Dysfunctional HDL as a therapeutic target for atherosclerosis prevention. *Current Medicinal Chemistry*.

[27] Cervellati C., Valacchi G., Tisato V., Zuliani G., Marsillach J. (2019). Evaluating the link between paraoxonase-1 levels and Alzheimer’s disease development. *Minerva Medica*.

[28] Marsillach J., Adorni M. P., Zimetti F., Papotti B., Zuliani G., Cervellati C. H. D. L. (2020). Proteome and Alzheimer’s disease: evidence of a link. *Antioxidants*.

[29] Ndrepepa G. (2019). Myeloperoxidase – a bridge linking inflammation and oxidative stress with cardiovascular disease. *Clinica Chimica Acta*.

[30] Green P. S., Mendez A. J., Jacob J. S. (2004). Neuronal expression of myeloperoxidase is increased in Alzheimer’s disease. *Journal of Neurochemistry*.

[31] Lefkowitz D. L., Mone J., Lefkowitz S. S. (2010). Myeloperoxidase: the good, the bad, and the ugly. *Current Immunology Reviews*.

[32] Carr A. C., McCall M. R., Frei B. (2000). Oxidation of LDL by myeloperoxidase and reactive nitrogen species: reaction pathways and antioxidant protection. *Arteriosclerosis, Thrombosis, and Vascular Biology*.

[33] Smallwood M. J., Nissim A., Knight A. R., Whiteman M., Haigh R., Winyard P. G. (2018). Oxidative stress in autoimmune rheumatic diseases. *Free Radical Biology & Medicine*.

[34] Trentini A., Rosta V., Spadaro S. (2020). Development, optimization and validation of an absolute specific assay for active myeloperoxidase (MPO) and its application in a clinical context: role of MPO specific activity in coronary artery disease. *Clinical Chemistry and Laboratory Medicine (CCLM)*.

[35] Breckwoldt M. O., Chen J. W., Stangenberg L. (2008). Tracking the inflammatory response in stroke in vivo by sensing the enzyme myeloperoxidase. *Proceedings of the National Academy of Sciences*.

[36] Üllen A., Singewald E., Konya V. (2013). Myeloperoxidase-derived oxidants induce blood-brain barrier dysfunction in vitro and in vivo. *PLoS One*.

[37] Volkman R., Ben-Zur T., Kahana A., Garty B. Z., Offen D. (2019). Myeloperoxidase deficiency inhibits cognitive decline in the 5XFAD mouse model of Alzheimer’s disease. *Frontiers in Neuroscience*.

[38] Fung K. Y., Wang C., Nyegaard S., Heit B., Fairn G. D., Lee W. L. (2017). SR-BI mediated transcytosis of HDL in brain microvascular endothelial cells is independent of caveolin, clathrin, and PDZK1. *Frontiers in Physiology*.

[39] Tzikas S., Schlak D., Sopova K. (2014). Increased myeloperoxidase plasma levels in patients with Alzheimer’s disease. *Journal of Alzheimer’s Disease*.

[40] Cheng Z., Yin J., Yuan H. (2018). Blood-derived plasma protein biomarkers for Alzheimer’s disease in Han Chinese. *Frontiers in Aging Neuroscience*.

[41] Marksteiner J., Imarhiagbe D., Defrancesco M., Deisenhammer E. A., Kemmler G., Humpel C. (2014). Analysis of 27 vascular-related proteins reveals that NT-proBNP is a potential biomarker for Alzheimer’s disease and mild cognitive impairment: a pilot-study. *Experimental Gerontology*.

[42] Wu C.-Y., Bawa K. K., Ouk M. (2020). Neutrophil activation in Alzheimer’s disease and mild cognitive impairment: a systematic review and meta-analysis of protein markers in blood and cerebrospinal fluid. *Ageing Research Reviews*.

[43] Koyama A., Okereke O. I., Yang T., Blacker D., Selkoe D. J., Grodstein F. (2012). Plasma amyloid-*β* as a predictor of dementia and cognitive decline. *Archives of Neurology*.

[44] Cao X., Chen P. (2020). Changes in serum amyloid A (SAA) and 8-OHdG in patients with senile early cognitive impairment. *Medical Science Monitor*.

[45] Shang J., Yamashita T., Fukui Y. (2018). Different associations of plasma biomarkers in Alzheimer’s disease, mild cognitive impairment, vascular dementia, and ischemic stroke. *Journal of Clinical Neurology*.

[46] van Oijen M., van der Meer I. M., Hofman A., Witteman J. C. M., Koudstaal P. J., Breteler M. M. B. (2006). Lipoprotein-associated phospholipase A2 is associated with risk of dementia. *Annals of Neurology*.

[47] Fitzpatrick A. L., Irizarry M. C., Cushman M., Jenny N. S., Chi G. C., Koro C. (2014). Lipoprotein-associated phospholipase A2 and risk of dementia in the Cardiovascular Health Study. *Atherosclerosis*.

[48] Bacchetti T., Vignini A., Giulietti A. (2015). Higher levels of oxidized low density lipoproteins in Alzheimer’s disease patients: roles for platelet activating factor acetyl hydrolase and paraoxonase-1. *Journal of Alzheimer’s Disease*.

[49] Doody R. S., Demirovic J., Ballantyne C. M. (2015). Lipoprotein-associated phospholipase A2, homocysteine, and Alzheimer’s disease. *Alzheimer’s & Dementia: Diagnosis, Assessment & Disease Monitoring*.

[50] van Himbergen T. M., Beiser A. S., Ai M. (2012). Biomarkers for insulin resistance and inflammation and the risk for all-cause dementia and Alzheimer disease: results from the Framingham Heart Study. *Archives of Neurology*.

[51] Ceballos-Picot I., Merad-Boudia M., Nicole A. (1996). Peripheral antioxidant enzyme activities and selenium in elderly subjects and in dementia of Alzheimer’s type--place of the extracellular glutathione peroxidase. *Free Radical Biology & Medicine*.

[52] Serra J. A., Domínguez R. O., Marschoff E. R., Guareschi E. M., Famulari A. L., Boveris A. (2009). Systemic oxidative stress associated with the neurological diseases of aging. *Neurochemical Research*.

[53] Casado Á., Encarnación López-Fernández M., Concepción Casado M., De La Torre R. (2008). Lipid peroxidation and antioxidant enzyme activities in vascular and Alzheimer dementias. *Neurochemical Research*.

[54] Rinaldi P., Polidori M. C., Metastasio A. (2003). Plasma antioxidants are similarly depleted in mild cognitive impairment and in Alzheimer’s disease. *Neurobiology of Aging*.

[55] Vural H., Demirin H., Kara Y., Eren I., Delibas N. (2010). Alterations of plasma magnesium, copper, zinc, iron and selenium concentrations and some related erythrocyte antioxidant enzyme activities in patients with Alzheimer’s disease. *Journal of Trace Elements in Medicine and Biology*.

[56] Leininger-Muller B., Hoy A., Herbeth B. (2003). Myeloperoxidase G-463A polymorphism and Alzheimer’s disease in the ApoEurope study. *Neuroscience Letters*.

[57] Ji W., Zhang Y. (2017). The association of MPO gene promoter polymorphisms with Alzheimer’s disease risk in Chinese Han population. *Oncotarget*.

[58] Zappia M., Manna I., Serra P. (2004). Increased risk for Alzheimer disease with the interaction of MPO and A2M polymorphisms. *Archives of Neurology*.

[59] Crawford F. C., Freeman M. J., Schinka J. A. (2001). Association between Alzheimer’s disease and a functional polymorphism in the myeloperoxidase gene. *Experimental Neurology*.

[60] Yu G., Liang Y., Huang Z., Jones D. W., Pritchard K. A. J., Zhang H. (2016). Inhibition of myeloperoxidase oxidant production by N-acetyl lysyltyrosylcysteine amide reduces brain damage in a murine model of stroke. *Journal of Neuroinflammation*.

[61] Shridas P., Tannock L. R. (2019). Role of serum amyloid A in atherosclerosis. *Current Opinion in Lipidology*.

[62] Gursky O. (2020). Structural basis for vital function and malfunction of serum amyloid A: an acute-phase protein that wears hydrophobicity on its sleeve. *Current Atherosclerosis Reports*.

[63] Uhlar C. M., Whitehead A. S. (1999). Serum amyloid A, the major vertebrate acute-phase reactant. *European Journal of Biochemistry*.

[64] Wilson P. G., Thompson J. C., Shridas P. (2018). Serum amyloid A is an exchangeable apolipoprotein. *Arteriosclerosis, Thrombosis, and Vascular Biology*.

[65] Cai X., Ahmad G., Hossain F. (2020). High-density lipoprotein (HDL) inhibits serum amyloid A (SAA)-induced vascular and renal dysfunctions in apolipoprotein E-deficient mice. *International Journal of Molecular Sciences*.

[66] Chang C., Pan Y., Du H., Wang X., Li X. (2020). Serum amyloid A1 can be a novel biomarker for evaluating the presence and severity of acute coronary syndrome. *Clinical Biochemistry*.

[67] Soria-Florido M. T., Castañer O., Lassale C. (2020). Dysfunctional high-density lipoproteins are associated with a greater incidence of acute coronary syndrome in a population at high cardiovascular risk: a nested case-control study. *Circulation*.

[68] Zhou J., Lu Y., Wang S., Chen K. (2020). Association between serum amyloid A levels and coronary heart disease: a systematic review and meta-analysis of 26 studies. *Inflammation Research*.

[69] Sack G. H. (2018). Serum amyloid A - a review. *Molecular Medicine*.

[70] Shridas P., De Beer M. C., Webb N. R. (2018). High-density lipoprotein inhibits serum amyloid A-mediated reactive oxygen species generation and NLRP3 inflammasome activation. *The Journal of Biological Chemistry*.

[71] Han C. Y., Tang C., Guevara M. E. (2016). Serum amyloid A impairs the antiinflammatory properties of HDL. *The Journal of Clinical Investigation*.

[72] Zimetti F., De Vuono S., Gomaraschi M. (2017). Plasma cholesterol homeostasis, HDL remodeling and function during the acute phase reaction. *Journal of Lipid Research*.

[73] Artl A., Marsche G., Lestavel S., Sattler W., Malle E. (2000). Role of serum amyloid A during metabolism of acute-phase HDL by macrophages. *Arteriosclerosis, Thrombosis, and Vascular Biology*.

[74] Chami B., Barrie N., Cai X. (2015). Serum amyloid A receptor blockade and incorporation into high-density lipoprotein modulates its pro-inflammatory and pro-thrombotic activities on vascular endothelial cells. *International Journal of Molecular Sciences*.

[75] Guo J.-T., Yu J., Grass D., de Beer F. C., Kindy M. S. (2002). Inflammation-dependent cerebral deposition of serum amyloid A protein in a mouse model of amyloidosis. *The Journal of Neuroscience*.

[76] Facci L., Barbierato M., Zusso M., Skaper S. D., Giusti P. (2018). Serum amyloid A primes microglia for ATP-dependent interleukin-1*β* release. *Journal of Neuroinflammation*.

[77] Jang S., Jang W. Y., Choi M. (2019). Serum amyloid A1 is involved in amyloid plaque aggregation and memory decline in amyloid beta abundant condition. *Transgenic Research*.

[78] Bowman G. L., Dayon L., Kirkland R. (2018). Blood-brain barrier breakdown, neuroinflammation, and cognitive decline in older adults. *Alzheimer’s & Dementia*.

[79] Matsumoto J., Dohgu S., Takata F. (2020). Serum amyloid A-induced blood-brain barrier dysfunction associated with decreased claudin-5 expression in rat brain endothelial cells and its inhibition by high-density lipoprotein in vitro. *Neuroscience Letters*.

[80] Miida T., Yamada T., Seino U. (2006). Serum amyloid A (SAA)-induced remodeling of CSF-HDL. *Biochimica et Biophysica Acta*.

[81] Vitali C., Wellington C. L., Calabresi L. (2014). HDL and cholesterol handling in the brain. *Cardiovascular Research*.

[82] Marchi C., Adorni M. P., Caffarra P. (2019). ABCA1- and ABCG1-mediated cholesterol efflux capacity of cerebrospinal fluid is impaired in Alzheimer’s disease. *Journal of Lipid Research*.

[83] Mullan R. H., McCormick J., Connolly M., Bresnihan B., Veale D. J., Fearon U. (2010). A role for the high-density lipoprotein receptor SR-B1 in synovial inflammation via serum amyloid-A. *The American Journal of Pathology*.

[84] D’Arrigo J. S. (2020). Biomimetic nanocarrier targeting drug(s) to upstream-receptor mechanisms in dementia: focusing on linking pathogenic cascades. *Biomimetics (Basel, Switzerland)*.

[85] Paterniti I., Cordaro M., Campolo M. (2014). Neuroprotection by association of palmitoylethanolamide with luteolin in experimental Alzheimer’s disease models: the control of neuroinflammation. *CNS & Neurological Disorders Drug Targets*.

[86] Barbierato M., Borri M., Facci L., Zusso M., Skaper S. D., Giusti P. (2017). Expression and differential responsiveness of central nervous system glial cell populations to the acute phase protein serum amyloid A. *Scientific Reports*.

[87] Kriska T., Marathe G. K., Schmidt J. C., McIntyre T. M., Girotti A. W. (2007). Phospholipase action of platelet-activating factor acetylhydrolase, but not paraoxonase-1, on long fatty acyl chain phospholipid hydroperoxides. *The Journal of Biological Chemistry*.

[88] Tellis C. C., Tselepis A. D. (2009). The role of lipoprotein-associated phospholipase A2 in atherosclerosis may depend on its lipoprotein carrier in plasma. *Biochimica et Biophysica Acta*.

[89] Hayek J., Cervellati C., Crivellari I., Pecorelli A., Valacchi G. (2017). Lactonase activity and lipoprotein-phospholipase A2 as possible novel serum biomarkers for the differential diagnosis of autism spectrum disorders and Rett syndrome: results from a pilot study. *Oxidative Medicine and Cellular Longevity*.

[90] Bonnefont-Rousselot D. (2016). Lp-PLA2, a biomarker of vascular inflammation and vulnerability of atherosclerosis plaques. *Annales Pharmaceutiques Françaises*.

[91] The Lp-PLA2 Studies Collaboration (2010). Lipoprotein-associated phospholipase A2 and risk of coronary disease, stroke, and mortality: collaborative analysis of 32 prospective studies. *The Lancet*.

[92] Lum H., Qiao J., Walter R. J. (2003). Inflammatory stress increases receptor for lysophosphatidylcholine in human microvascular endothelial cells. *American Journal of Physiology-Heart and Circulatory Physiology*.

[93] Huang F., Subbaiah P. V., Holian O. (2005). Lysophosphatidylcholine increases endothelial permeability: role of PKC*α* and RhoA cross talk. *American Journal of Physiology-Lung Cellular and Molecular Physiology*.

[94] Romero J. R., Preis S. R., Beiser A. S. (2012). Lipoprotein phospholipase A2 and cerebral microbleeds in the Framingham Heart Study. *Stroke*.

[95] Davidson J. E., Lockhart A., Amos L. (2012). Plasma lipoprotein-associated phospholipase A2 activity in Alzheimer’s disease, amnestic mild cognitive impairment, and cognitively healthy elderly subjects: a cross-sectional study. *Alzheimer’s Research & Therapy*.

[96] Cai R., Huang R., Han J. (2017). Lipoprotein-associated phospholipase A2 is associated with risk of mild cognitive impairment in Chinese patients with type 2 diabetes. *Scientific Reports*.

[97] Acharya N. K., Levin E. C., Clifford P. M. (2013). Diabetes and hypercholesterolemia increase blood-brain barrier permeability and brain amyloid deposition: beneficial effects of the LpPLA2 inhibitor darapladib. *Journal of Alzheimer’s Disease*.

[98] Maher-Edwards G., De’Ath J., Barnett C., Lavrov A., Lockhart A. (2015). A 24-week study to evaluate the effect of rilapladib on cognition and cerebrospinal fluid biomarkers of Alzheimer’s disease. *Alzheimer's & Dementia: Translational Research & Clinical Interventions*.

[99] Lubos E., Loscalzo J., Handy D. E. (2011). Glutathione peroxidase-1 in health and disease: from molecular mechanisms to therapeutic opportunities. *Antioxidants & Redox Signaling*.

[100] Zhang Y., Roh Y. J., Han S.-J. (2020). Role of selenoproteins in redox regulation of signaling and the antioxidant system: a review. *Antioxidants*.

[101] Brigelius-Flohé R., Maiorino M. (2013). Glutathione peroxidases. *Biochimica et Biophysica Acta (BBA) - General Subjects*.

[102] Brites F., Martin M., Guillas I., Kontush A. (2017). Antioxidative activity of high-density lipoprotein (HDL): mechanistic insights into potential clinical benefit. *BBA Clinical*.

[103] Caroline C., Worley B. L., Phaëton R., Hempel N. (2020). Extracellular glutathione peroxidase GPx3 and its role in cancer. *Cancers*.

[104] Buijsse B., Lee D.-H., Steffen L. (2012). Low serum glutathione peroxidase activity is associated with increased cardiovascular mortality in individuals with low HDLc’s. *PLoS One*.

[105] Pastori D., Pignatelli P., Farcomeni A. (2016). Aging-related decline of glutathione peroxidase 3 and risk of cardiovascular events in patients with atrial fibrillation. *Journal of the American Heart Association*.

[106] Ballatori N., Krance S. M., Marchan R., Hammond C. L. (2009). Plasma membrane glutathione transporters and their roles in cell physiology and pathophysiology. *Molecular Aspects of Medicine*.

[107] Björnstedt M., Xue J., Huang W., Åkesson B., Holmgren A. (1994). The thioredoxin and glutaredoxin systems are efficient electron donors to human plasma glutathione peroxidase. *The Journal of Biological Chemistry*.

[108] Bermingham E. N., Hesketh J. E., Sinclair B. R., Koolaard J. P., Roy N. C. (2014). Selenium-enriched foods are more effective at increasing glutathione peroxidase (GPx) activity compared with selenomethionine: a meta-analysis. *Nutrients*.

[109] Fairweather-Tait S. J., Bao Y., Broadley M. R. (2011). Selenium in human health and disease. *Antioxidants & Redox Signaling*.

[110] Chang Y.-T., Chang W.-N., Tsai N.-W. (2014). The roles of biomarkers of oxidative stress and antioxidant in Alzheimer’s disease: a systematic review. *BioMed Research International*.

[111] Fernandes M. A., Proenca M. T., Nogueira A. J. (1999). Influence of apolipoprotein E genotype on blood redox status of Alzheimer’s disease patients. *International Journal of Molecular Medicine*.

[112] Padurariu M., Ciobica A., Hritcu L., Stoica B., Bild W., Stefanescu C. (2010). Changes of some oxidative stress markers in the serum of patients with mild cognitive impairment and Alzheimer’s disease. *Neuroscience Letters*.

[113] Torres L. L., Quaglio N. B., de Souza G. T. (2011). Peripheral oxidative stress biomarkers in mild cognitive impairment and Alzheimer’s disease. *Journal of Alzheimer’s Disease*.

[114] Rothem L., Hartman C., Dahan A., Lachter J., Eliakim R., Shamir R. (2007). Paraoxonases are associated with intestinal inflammatory diseases and intracellularly localized to the endoplasmic reticulum. *Free Radical Biology & Medicine*.

[115] Schweikert E.-M., Devarajan A., Witte I. (2012). PON3 is upregulated in cancer tissues and protects against mitochondrial superoxide-mediated cell death. *Cell Death and Differentiation*.

[116] Marsillach J., Mackness B., Mackness M. (2008). Immunohistochemical analysis of paraoxonases-1, 2, and 3 expression in normal mouse tissues. *Free Radical Biology & Medicine*.

[117] Draganov D. I., Teiber J. F., Speelman A., Osawa Y., Sunahara R., La Du B. N. (2005). Human paraoxonases (PON1, PON2, and PON3) are lactonases with overlapping and distinct substrate specificities. *Journal of Lipid Research*.

[118] Ng C. J., Bourquard N., Hama S. Y. (2007). Adenovirus-mediated expression of human paraoxonase 3 protects against the progression of atherosclerosis in apolipoprotein E-deficient mice. *Arteriosclerosis, Thrombosis, and Vascular Biology*.

[119] Shih D. M., Yu J. M., Vergnes L. (2015). PON3 knockout mice are susceptible to obesity, gallstone formation, and atherosclerosis. *The FASEB Journal*.

[120] Teiber J. F., Horke S., Haines D. C. (2008). Dominant role of paraoxonases in inactivation of the Pseudomonas aeruginosa quorum-sensing signal N-(3-oxododecanoyl)-L-homoserine lactone. *Infection and Immunity*.

[121] Robertson K. S., Hawe E., Miller G. J., Talmud P. J., Humphries S. E., Northwick Park Heart Study II (2003). Human paraoxonase gene cluster polymorphisms as predictors of coronary heart disease risk in the prospective Northwick Park Heart Study II. *Biochimica et Biophysica Acta*.

[122] Campo S., Sardo A. M., Campo G. M. (2004). Identification of paraoxonase 3 gene (PON3) missense mutations in a population of southern Italy. *Mutation Research*.

[123] Erlich P. M., Lunetta K. L., Cupples L. A. (2006). Polymorphisms in the PON gene cluster are associated with Alzheimer disease. *Human Molecular Genetics*.

[124] Carlson C. S., Heagerty P. J., Hatsukami T. S. (2006). TagSNP analyses of the PON gene cluster: effects on PON1 activity, LDL oxidative susceptibility, and vascular disease. *Journal of Lipid Research*.

[125] García-Heredia A., Marsillach J., Aragonès G. (2011). Serum paraoxonase-3 concentration is associated with the severity of hepatic impairment in patients with chronic liver disease. *Clinical Biochemistry*.

[126] Aragonès G., García-Heredia A., Guardiola M. (2012). Serum paraoxonase-3 concentration in HIV-infected patients. Evidence for a protective role against oxidation. *Journal of Lipid Research*.

[127] Rull A., García R., Fernández-Sender L. (2012). Serum paraoxonase-3 concentration is associated with insulin sensitivity in peripheral artery disease and with inflammation in coronary artery disease. *Atherosclerosis*.

[128] Sans T., Rull A., Luna J. (2012). Monocyte chemoattractant protein-1 and paraoxonase-1 and 3 levels in patients with sepsis treated in an intensive care unit: a preliminary report. *Clinical Chemistry and Laboratory Medicine*.

[129] Marsillach J., Becker J. O., Vaisar T. (2015). Paraoxonase-3 is depleted from the high-density lipoproteins of autoimmune disease patients with subclinical atherosclerosis. *Journal of Proteome Research*.

[130] Costa L. G., Giordano G., Furlong C. E. (2011). Pharmacological and dietary modulators of paraoxonase 1 (PON1) activity and expression: the hunt goes on. *Biochemical Pharmacology*.

[131] Furlong C. E., Marsillach J., Jarvik G. P., Costa L. G. (2016). Paraoxonases-1, -2 and -3: what are their functions?. *Chemico-Biological Interactions*.

[132] Zaplatic E., Bule M., Shah S. Z. A., Uddin M. S., Niaz K. (2019). Molecular mechanisms underlying protective role of quercetin in attenuating Alzheimer’s disease. *Life Sciences*.

[133] Costa L. G., Garrick J. M., Roquè P. J., Pellacani C. (2016). Mechanisms of neuroprotection by quercetin: counteracting oxidative stress and more. *Oxidative Medicine and Cellular Longevity*.

[134] Qi Y., Guo L., Jiang Y., Shi Y., Sui H., Zhao L. (2020). Brain delivery of quercetin-loaded exosomes improved cognitive function in AD mice by inhibiting phosphorylated tau-mediated neurofibrillary tangles. *Drug Delivery*.

